# SWI/SNF regulates the alternative processing of a specific subset of pre-mRNAs in *Drosophila melanogaster*

**DOI:** 10.1186/1471-2199-12-46

**Published:** 2011-11-02

**Authors:** Johan Waldholm, Zhi Wang, David Brodin, Anu Tyagi, Simei Yu, Ulrich Theopold, Ann Kristin Östlund Farrants, Neus Visa

**Affiliations:** 1 Department of Molecular Biology and Functional Genomics, Stockholm University, SE-10691 Stockholm, Sweden; 2 Bioinformatics and Expression Analysis Core Facility, Department of Biosciences and Nutrition, Karolinska Institutet, SE-14157 Huddinge, Sweden; 3 Wenner-Gren Institute, Stockholm University, SE-10691 Stockholm, Sweden; 4 Department of Biochemistry, University of Würzburg, Am Hubland, D-97074 Würzburg, Germany

## Abstract

**Background:**

The SWI/SNF chromatin remodeling factors have the ability to remodel nucleosomes and play essential roles in key developmental processes. SWI/SNF complexes contain one subunit with ATPase activity, which in *Drosophila melanogaster *is called Brahma (Brm). The regulatory activities of SWI/SNF have been attributed to its influence on chromatin structure and transcription regulation, but recent observations have revealed that the levels of Brm affect the relative abundances of transcripts that are formed by alternative splicing and/or polyadenylation of the same pre-mRNA.

**Results:**

We have investigated whether the function of Brm in pre-mRNA processing in *Drosophila melanogaster *is mediated by Brm alone or by the SWI/SNF complex. We have analyzed the effects of depleting individual SWI/SNF subunits on pre-mRNA processing throughout the genome, and we have identified a subset of transcripts that are affected by depletion of the SWI/SNF core subunits Brm, Snr1 or Mor. The fact that depletion of different subunits targets a subset of common transcripts suggests that the SWI/SNF complex is responsible for the effects observed on pre-mRNA processing when knocking down Brm. We have also depleted Brm in larvae and we have shown that the levels of SWI/SNF affect the pre-mRNA processing outcome *in vivo*.

**Conclusions:**

We have shown that SWI/SNF can modulate alternative pre-mRNA processing, not only in cultured cells but also *in vivo*. The effect is restricted to and specific for a subset of transcripts. Our results provide novel insights into the mechanisms by which SWI/SNF regulates transcript diversity and proteomic diversity in higher eukaryotes.

## Background

ATP-dependent chromatin remodeling factors play key roles in eukaryotic genome regulation. The remodeling factors affect a diverse subset of cellular processes through their ability to influence the chromatin structure [reviewed in 1]. The SWI/SNF chromatin remodeling factors are dynamic multiprotein complexes that have the ability to remodel nucleosomes preferentially at the promoter regions of genes. The SWI/SNF complexes are recruited to their substrates by acetylated histone tails, and they use energy from ATP hydrolysis to modify DNA-protein contacts in the nucleosome. Their activities modulate the accessibility of regulatory sequences in the DNA. The outcome of such a regulation is either the activation or the repression of gene expression, depending on the genomic context and on the binding of specific co-regulators [reviewed in 2].

Chromatin remodeling complexes contain one subunit with ATPase activity. In humans, the catalytic ATPase subunit of the SWI/SNF complex is either the Brahma protein (hBrm) or the Brahma-related gene 1 protein (Brg1), whereas in *Drosophila melanogaster *there is a unique ATPase subunit, Brm. Brm, together with Moira (Mor) and Snf5-related 1 (Snr1), forms the core of the SWI/SNF complex in *Drosophila*. Additionally, a multitude of signature subunits associate with the SWI/SNF core and define functionally distinct SWI/SNF complexes, for instance BAP (for "Brahma-associated proteins") and PBAP (for "Polybromo-associated BAP") [3-6]. The signature subunit Osa is found in BAP, while BAP170, Polybromo (PB) and Syap define the PBAP complex [3,7].

The SWI/SNF complexes play essential roles in key developmental processes [8-10]. The hBrm and Brg1 proteins are required for tissue-specific gene expression and cell cycle progression [11]. In flies, SWI/SNF is needed for cell growth and survival in the wing imaginal disc [6], photoreceptor differentiation [12], immune system function [5], cell progression through mitosis [4] and normal embryonic segmentation [13,14].

The regulatory activities of SWI/SNF have been attributed to its effect on chromatin structure and transcription regulation, but a number of recent observations (see below) have revealed that the SWI/SNF complex is also implicated in the regulation of the alternative processing of precursor mRNAs (pre-mRNAs). In eukaryotic cells, the pre-mRNA is processed through capping of the 5' end, splicing, and formation of the 3' end. Capping is a very early event that occurs shortly after the RNA polymerase II (RNAP II) leaves the promoter [15]. Splicing is often co-transcriptional and takes place during transcription elongation, although it can also occur post-transcriptionally [16,17]. In the last step of pre-mRNA maturation, the pre-mRNA is cleaved approximately 25 nt downstream of the polyadenylation signal, and the 3' end of the transcript is polyadenylated [18]. Most transcripts synthesized by RNAP II undergo the above-mentioned RNA processing events, and pre-mRNA processing is usually regulated. Different types of factors determine the choice of exons incorporated into the mature mRNA, a process known as "alternative splicing". The usage of alternative cleavage and polyadenylation sites is also regulated. Recent estimates indicate that approximately 95% of the human pre-mRNAs experience alternative processing events, which provides a substantial source of protein variability [19,20]. In *Drosophila melanogaster*, over 40% of the genes show multiple patterns of expression that suggest alternative processing or alternative promoter usage [21].

Batsche and coworkers [22] showed that expression of hBrm in HeLa cells favored the inclusion of variable exons in the CD44 mRNA, and proposed a kinetic model in which hBrm reduces the elongation rate of RNAP II over the CD44 variable exons, providing time for spliceosome assembly at the proximal, weak splice sites of the CD44 pre-mRNA. The influence of the RNAP II elongation rate on the choice of alternative splice sites had been previously shown for both human and *Drosophila *genes [23]. Interestingly, expression of mutated hBrm with an inactive ATPase domain also promotes inclusion of the CD44 variable exons, which suggests that the function of hBrm in this context is independent of chromatin remodelling. Studies on the expression of the telomerase reverse transcriptase (TERT) gene in NCI-H1299 cells have revealed alternative inclusion of exons 7 and 8 in the presence of hBrm/Brg1 [24]. Further, immuno-electron microscopy studies by Tyagi and coworkers [25] showed that Brm is associated with nascent Balbiani ring (BR) pre-mRNP particles in the salivary glands of *Chironomus tentans*. Cell fractionation studies confirmed the mRNP interaction of Brm in both *Drosophila melanogaster *S2 cells and human HeLa cells. In addition, Tyagi and coworkes [25] observed that depletion of dBrm by RNA interference (RNAi) in S2 cells affects alternative pre-mRNA splicing and polyadenylation decisions. In summary, the cellular levels of Brm affect the levels of certain mRNAs, and Brm participates in the regulation of alternative pre-mRNA processing in addition to its role in chromatin remodeling. The mechanisms by which Brm affects the processing of some pre-mRNAs are far from being understood. We have investigated whether the function of Brm in pre-mRNA processing is mediated by Brm alone or by the SWI/SNF complex. We have analyzed the effects of depleting other SWI/SNF subunits on pre-mRNA processing genome-wide, and we present evidence that other subunits in addition to Brm are involved in the regulation of pre-mRNA splicing and/or polyadenylation. We have also depleted Brm in flies and we have shown that SWI/SNF levels affect pre-mRNA processing *in vivo*.

## Results

### Depletion of SWI/SNF subunits affects the relative levels of alternatively processed transcripts

**Figure 1:**
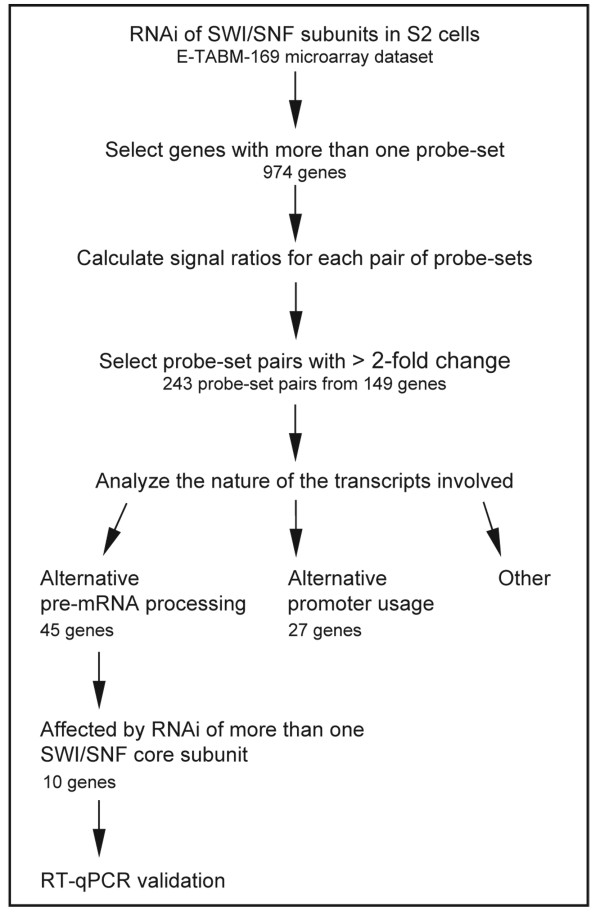
**Work-flow for the analysis of microarray data**. Description of the analysis of gene expression microarray data to extract information on alternative pre-mRNA processing. For details, see the Materials and Methods section.

Depletion of Brm by RNAi affects the alternative processing of a number of pre-mRNAs in S2 cells of *D. melanogaster *[25]. We have studied the role of other SWI/SNF subunits in pre-mRNA processing by a genome-wide analysis in which we looked for genes with pre-mRNA processing patterns that were affected by depletion of individual SWI/SNF subunits. We used data from the E-TABM-169 experiments http://www.ebi.ac.uk/microarray-as/aew/ from Moshkin and coworkers [4]. Moshkin and coworkers determined the expression profiles of S2 cells after depletion of individual SWI/SNF subunits by RNAi and microarray hybridization using the Affymetrix *Drosophila *Genome 2 arrays. These arrays are not particularly designed as splicing arrays and do not cover the entire transcriptome, but they provide information about a large number of pre-mRNA processing events. We mined the TABM-169 data and selected the genes represented in the array by more than one probe set (974 genes). We excluded from the analysis those probe sets that showed large signal variations among individual experiments (those for which the ratio of average to standard deviation was less than 1.25 were excluded). For each gene, we calculated the ratios between probe sets, and we compared the probe-set ratios between control cells (*mock *RNAi) and RNAi-treated cells (Figure 1). We selected the probe-set pairs for which the ratio had changed by more than a factor of two. We analyzed cells treated by RNAi for Brm, Mor, Snr1, PB, Bap170 and Osa using the same type of analysis. This gave a total of 243 probe-set pairs, corresponding to 149 genes (15% of the total number of genes included in the analysis) that showed changed ratios upon depletion of at least one SWI/SNF subunit. We then used the gene models available at Flybase http://flybase.org/ and the probe-set annotations at Affymetrix http://www.affymetrix.com/analysis/index.affx to analyze the structure of the 149 selected genes and the nature of the events modified by depletion of SWI/SNF subunits. In some cases, the multiple probe sets targeted different parts of the same transcript, pseudogenes or alternative transcripts derived from alternative promoters of the same gene. Probe sets targeting multiple transcripts were discarded. For 45 genes, we established unambiguously that the changed ratios between transcripts were due to differences in the abundances of alternatively processed transcripts (4.6% of the total number of genes included in the analysis) (Additional file 1, Table S1). We also identified 27 genes (Additional file 1, Table S2) that showed changed promoter usage upon SWI/SNF depletion, as expected based on the role of SWI/SNF in transcription regulation. One of these 27 genes was Eip74EF, an ecdysone-inducible transcription factor. This observation agrees with the results of a previous study in which hormone-response genes were identified as direct regulatory targets of Brahma *in vivo *[26].

An analysis of gene ontology (GO) terms revealed functional differences between the group of genes affected at the level of alternative promoter usage (genes listed in Table S2) and the groups of genes affected at the level of regulation of alternative pre-mRNA processing (genes listed in Table S1). The GO terms related to cell cycle regulation and dorso-ventral pattern formation were enriched for the genes whose promoter usage was affected by depletion of SWI/SNF subunits. The terms enriched in the group of genes that were affected at the level of alternative pre-mRNA processing were instead related to eye development and RNA metabolic processes (Table 1). The differences in GO-term enrichment suggest that SWI/SNF acts through different mechanisms on specific subsets of genes.

**Table 1:** Gene ontology analysis of genes affected by depletion of SWI/SNF in S2 cells

Genes with pre-mRNA processing affected			
**GO ID**	**Term**	**Log (odds ratio)**	**p**

0009790	embryo development	2.33	0.0088
0009887	organ morphogenesis	2.75	0.0010
0048513	organ development	2.11	0.0032
0001745	compound eye morphogenesis	3.14	0.0059
0007601	visual perception	3.60	0.0098
0050953	sensory perception of light stimulus	3.59	0.0098
0051252	regulation of RNA metabolic process	2.06	0.0098
**Genes with promoter usage affected**			
**GO ID**	**Term**	**Log (odds ratio)**	**p**

0022402	cell cycle process	2.76	0.0045
0000279	M phase	3.00	0.0034
0000022	mitotic spindle elongation	4.51	0.0021
0007052	mitotic spindle organization	3.33	0.0079
0000226	microtubule cytoskeleton organization	3.92	< 0.0001
0048513	organ development	2.25	0.0043
0035220	wing disc development	3.22	0.0043
0048190	wing disc dorsal/ventral pattern formation	5.01	0.0011
0009165	nucleotide biosynthetic process	3.50	0.0060
0003735	structural constituent of ribosome	3.16	0.0098
0006996	organelle organization	2.24	0.0043
0065008	regulation of biological quality	2.37	0.0096

The table shows the results from GO-enrichment analysis for two separate groups of genes, as indicated. The table shows terms that include more than two hits, that show a log (odds ratio) > 2 (odds ratio > 7.3) and p < 0.01 (hypergeometric test).

**Figure 2:**
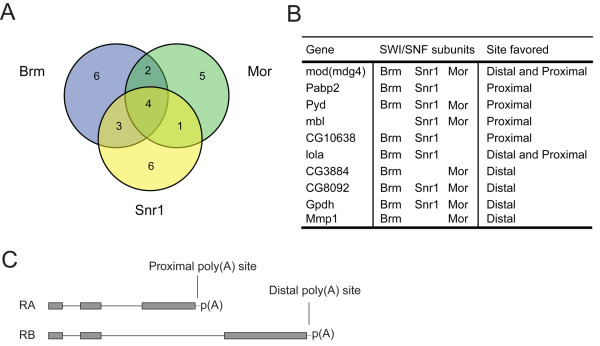
**Genome-wide identification of pre-mRNA processing events affected by depletion of individual SWI/SNF core subunits in S2 cells**. (A) Venn diagram of the extent of overlap of the genes affected by each individual SWI/SNF core subunit. (B) Genes affected by depletion of more than one core subunit. For each gene, the processing site favored by the depletion, proximal or distal, is indicated. (C) Schematic representation of two alternative transcripts, RA and RB, from a hypothetical gene showing mutually exclusive splice and polyadenylation sites. The grey boxes represent exons. The thin lines represent introns.

Among the 45 genes that displayed changed abundances of alternatively processed transcripts upon SWI/SNF depletion, we identified 15 genes that were affected by the knockdown of Brm, 14 genes affected by the knockdown of Snr1, and 12 genes affected by the knockdown of Mor. We also found a group of ten genes that were sensitive to the depletion of at least two SWI/SNF core subunits, and four genes affected by depletion of any of the three core subunits (Figures 2A, B). Random samplings for each of the core subunits from the 45 selected genes were subjected to 10 millions iterations to investigate whether the overlap found for all three subunits was higher than expected by random. The result showed that the probability of finding at least 4 genes in all three subgroups randomly is < 0.023.

The depletion of the individual signature subunits of the SWI/SNF complex - BAP170, PB or OSA - did not produce statistically significant effects on pre-mRNA splicing genome-wide, but the simultaneous depletion of the three signature subunits often mimicked the effects of depletion of individual core subunits (Additional file 2, Figure S1).

We analyzed the structure of the transcripts affected by the SWI/SNF depletions on pre-mRNA processing and found that the depletion of SWI/SNF subunits affected not only the alternative splicing of the primary transcripts but also the use of alternative polyadenylation sites. In most cases, the affected event was a choice between mutually exclusive splice sites and polyadenylation sites. Such alternative splicing-polyadenylation events are illustrated in Figure 2C.

Recent studies in human cells have led to the proposal that Brm can reduce the elongation rate of RNA polymerase II and thereby affect splice-site selection by promoting the usage of proximal sites [22]. A similar mechanism might function in *Drosophila *cells. If so, depletion of Brm is expected to increase the use of distal sites. We analyzed whether the changes observed in the ten selected genes shown in Figure 2B followed a defined trend in terms of proximal or distal usage. The use of the distal sites was indeed favored in some cases. However, the use of a proximal processing site was increased following SWI/SNF depletion in other cases. This suggests that mechanisms additional to the one described above contribute to the effects of SWI/SNF on pre-mRNA processing (see Discussion).

### Validation of the microarray results in S2 cells

**Figure 3:**
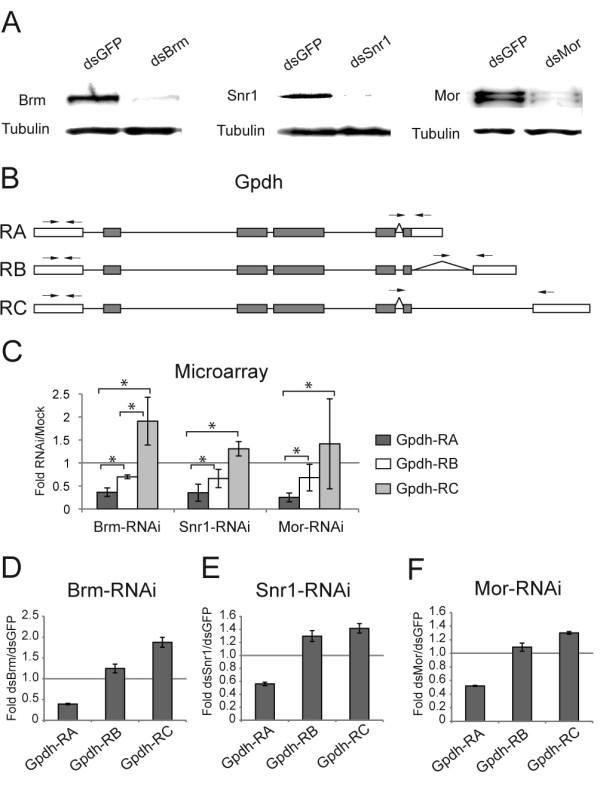
**Depletion of the SWI/SNF core subunits changes the alternative processing of the Gpdh pre-mRNA in S2 cells**. S2 cells were treated with dsRNA for Brm, Snr1 or Mor. Mock-treated cells were used as controls for microarray experiments. The control cells in the validation experiments were treated with GFP-dsRNA. (A) The effects of the dsRNA treatments analyzed by Western blotting. (B) The Gpdh mRNAs. The boxes and the thin lines represent exons and introns, respectively. White and dark boxes represent untranslated regions and coding sequences, respectively. The primers used for qPCR are indicated by small arrows on top of each transcript. (C) Results from the microarray data. The histograms show the average ratios between the abundance of each isoform in RNAi-treated cells compared to mock-treated cells (based on data from E-TABM-169). The bars represent average ratios from four mock replicates and three replicates for each of the depletion experiments, respectively. The error bars represent standard deviations. The stars mark transcript ratios that were affected by the depletions according to the selection criteria described in the text. (D), (E) and (F) Validation of the effects of the depletions. Either Brm, Snr1 or Mor was knocked down in S2 cells, and the relative abundance of each transcript was quantified by RT-qPCR. The transcript-specific primer pairs target exon-exon junctions and downstream exons. The abundance of each transcript was calculated relative to the abundance of the first, constitutive exon. The results are expressed as the factor change between RNAi-treated and GFP-treated cells. The bars represent averages from three technical replicates from an RNAi experiment. The error bars represent standard deviations.

**Figure 4:**
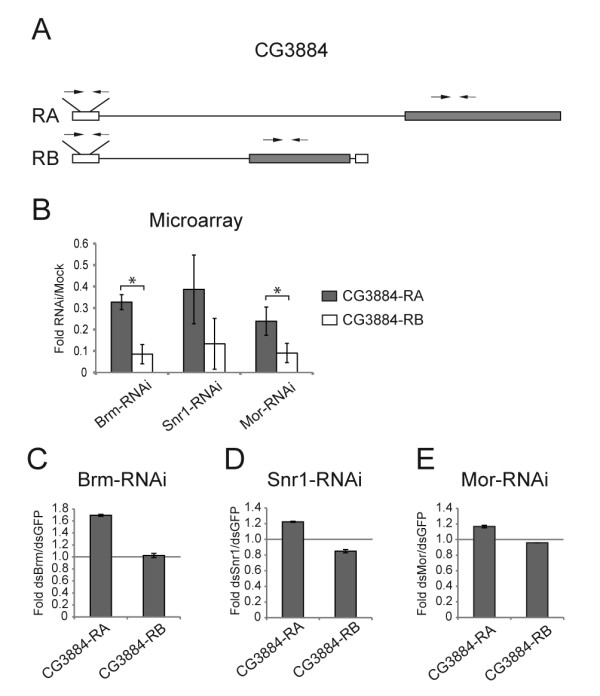
**Depletion of the SWI/SNF core subunits changes the alternative processing of the CG3884 pre-mRNA in S2 cells**. S2 cells were treated with dsRNA for either Brm, Snr1 or Mor. Mock-treated cells were used as controls for microarray experiments (Moshkin et al. 2007), whereas the control cells in the validation experiments were treated with GFP-dsRNA. (A) The exon-intron organization of the two CG3884 mRNAs. The positions of the primer pairs used for qPCR are indicated by small arrows on top of each transcript. (B) Summary of the results derived from the microarray data. For each of the SWI/SNF core subunits, the histogram shows the average ratios between the abundance of each isoform in RNAi-treated cells compared to mock-treated cells (base on data from E-TABM-169). The bars represent average ratios calculated as in Figure 3. The error bars represent standard deviations calculated from six mock replicates and four replicates for each of the depletion experiments, respectively. The stars mark transcript ratios that were affected by depletion of Brm, Snr1 or Mor according to the selection criteria described in the main text. (C), (D) and (E) Validation of the effects of the depletions on the relative abundances of the CG3882 mRNAs. Either Brm, Snr1 or Mor was knocked down in S2 cells, and the relative abundance of each transcript was quantified by RT-qPCR using the primer pairs indicated in (A). The abundance of each alternative transcript was calculated relative to the abundance of the first, constitutive exon, as in Figure 3.

**Figure 5:**
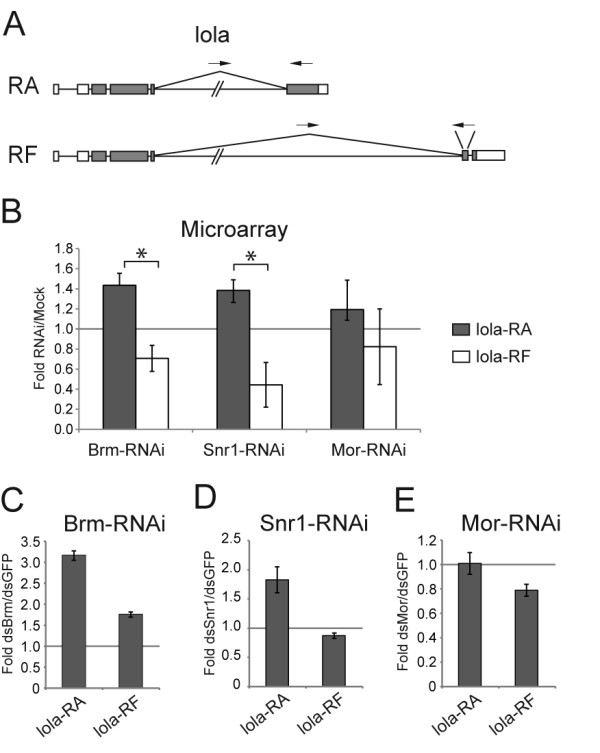
**Depletion of the SWI/SNF core subunits changes the relative abundances of some lola mRNAs in S2 cells**. S2 cells were treated with dsRNA for either Brm, Snr1 or Mor. Mock-treated cells were used as controls for microarray experiments (Moshkin et al. 2007), whereas the control cells in the validation experiments were treated in parallel with dsRNA for GFP. (A) The exon-intron organization of two lola mRNAs. The positions of the primer pairs used for qPCR are indicated by small arrows on top of each transcript. (B) Summary of the results derived from the microarray data. For each of the SWI/SNF core subunits, the histogram shows the average ratios between the abundance of each isoform in RNAi-treated cells compared to mock-treated cells (base on data from E-TABM-169). The bars represent average ratios calculated as in Figure 3. The error bars represent standard deviations. The stars mark transcript ratios that were affected by depletion of Brm, Snr1 or Mor according to the selection criteria described in the main text. (C), (D) and (E) Validation of the effects of the depletions on the relative abundances of the lola mRNAs. Either Brm, Snr1 or Mor was knocked down in S2 cells, and the relative abundance of each transcript was quantified by RT-qPCR as in Figure 3. The primer pairs used for the quantification target exon-exon junctions and downstream exons, as indicated by small arrows in (A). The abundances of the lola-RA and lola-RF transcripts were calculated relative to the abundance of the Act5C mRNA in the same samples.

**Figure 6:**
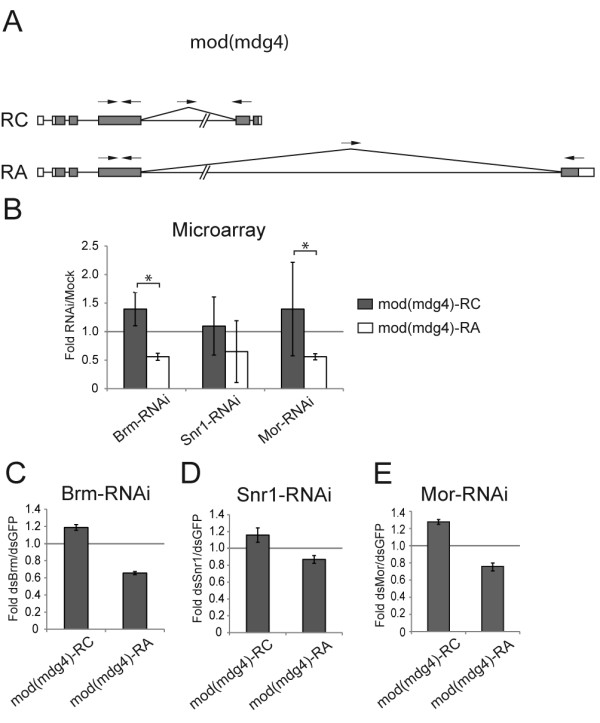
**Depletion of the SWI/SNF core subunits changes the relative abundances of some mod(mdg4) mRNAs in S2 cells**. S2 cells were treated with dsRNA for either Brm, Snr1 or Mor. Mock-treated cells were used as controls for microarray experiments (Moshkin et al. 2007), whereas the control cells in the validation experiments were treated in parallel with dsRNA for GFP. (A) The exon-intron organization of two mod(mdg4) mRNAs. The positions of the primer pairs used for qPCR are indicated by small arrows on top of each transcript. (B) Summary of the results derived from the microarray data. For each of the SWI/SNF core subunits, the histogram shows the average ratios between the abundance of each isoform in RNAi-treated cells compared to mock-treated cells (base on data from E-TABM-169). The bars represent average ratios calculated as in Figure 3. The error bars represent standard deviations. The stars mark transcript ratios that were affected by depletion of Brm, Snr1 or Mor according to the selection criteria described in the main text. (C), (D) and (E) Validation of the effects of the depletions on the relative abundances of the mod(mdg4) mRNAs, as in Figure 3. Either Brm, Snr1 or Mor was knocked down in S2 cells, and the relative abundance of each transcript was quantified by RT-qPCR using the primer pairs that target exon-exon junctions and downstream exons, as indicated in (A). The abundances of mod(mdg4)-RA and mod(mdg4)-RC were calculated relative to the abundance of a constitutive exon, as indicated in (A).

The results described above are based on the use of gene expression arrays that targeted specific exons. We next validated the microarray results described above for four selected genes: *Gpdh*, *CG3884*, *mod(mdg4) *and *lola *(Additional file 1, Table S3). The validation results are shown in Figures 3, 4, 5, 6. The figures show data for all the analyzed transcripts, but not all the ratios among the transcripts of a given gene were affected by SWI/SNF depletion according to the selection criteria described above. For each validated gene, the transcript ratios that were affected by depletion of Brm, Mor or Snr1 are marked with stars in the figure.

We knocked down either Brm, Mor or Snr1 in S2 cells by RNAi, and we analyzed the effects of the knock-down treatments on the processing of the target transcripts by RT-qPCR. The efficiencies of the knock-down treatments were assessed by Western blotting (Figure 3A). For each of the genes, we designed primers aimed at detecting the alternatively processed transcripts. The primers targeted specific exons and exon-exon junctions. Figure 3 shows the results of the analysis of the *Gpdh *pre-mRNA processing. The microarray data show that depletion of either Brm, Mor or Snr1 results in decreased *Gpdh*-RA levels and increased levels of *Gpdh*-RC (Figure 3C). We sought to validate these microarray observations. For comparative purposes, we measured the levels of each alternative transcript by RT-qPCR, and we expressed the levels of each alternative transcript relative to the levels of the first exon, which is constitutive. Normalizing the data against the levels of a constitutive exon reveals the relative differences between isoforms and eliminates influences due to changes in transcription. Figures 3D, E, F show that the observations derived from the microarray analysis could be validated.

The analysis of the *CG3884 *pre-mRNA processing is shown in Figure 4. The microarray data had shown that depletion of SWI/SNF subunits reduced the overall levels of *CG3884 *expression and favored the formation of the RA transcript compared to RB (Figure 4B). The prevalence of the RA transcript was particularly significant after Brm depletion (see the large changes for Brm-RNAi in Figure 4B). Our validation experiments confirmed that depletion of Brm favors the formation of the RA isoform (Figure 4C). The level of each alternative transcript was normalised relative to the level of an upstream constitutive exon (see the position of primer pairs in Figure 4B) to avoid the influence of the overall *CG3884 *expression level and to measure exclusively the effects on alternative processing. Depletion of either Snr1 or Mor (Figures 4D, E) also showed a certain tendency towards the formation of RA transcripts, but the magnitude of the effects was less pronounced, in agreement with the microarray data.

Processing of the *lola *transcripts was also affected by depletion of SWI/SNF subunits. The annotations in FlyBase state that the *lola *gene (CG12052) has 26 different transcripts and encodes for 26 different polypeptides. This variation is achieved through alternative promoter usage, alternative splicing and alternative polyadenylation. The microarray analysis revealed that the relative abundances of specific *lola *isoforms were sensitive to the levels of SWI/SNF. Figure 5 shows the analysis of the expression of the *lola*-RA and *lola*-RF transcripts, two transcripts that originate from the same pre-mRNA (i.e. from the same promoter). The microarray experiments showed that depletion of either Brm or Snr1 resulted in increased levels of RA transcripts and reduced levels of RF transcripts, whereas depletion of Mor did not have any significant effect. We analyzed the expression of these two isoforms in S2 cells upon depletion of Brm, Snr1 or Mor, and we quantified the transcript levels by RT-qPCR, as above. In the case of the *lola *analysis, the transcript levels were normalized to the levels of a house-keeping RNA, *Act5C*, because the constitutive *lola *exons are present in a large variety of transcripts that originate from at least three different promoters. Figures 5C, D, E show that our validation experiments reproduced the microarray data and confirmed that depletions of the SWI/SNF subunits Brm and Snr1 promote the formation of certain *lola *isoforms, in this case RA.

The fourth gene that we analyzed was *mod(mdg4)*, also a gene with a highly complex pattern of expression. There are 29 annotated transcripts and 29 annotated polypeptides for *mod(mdg4)*. Figure 6 summarizes the effects of SWI/SNF depletion on the levels of the RC and RA isoforms according to the microarray analysis, and shows that depletion of either Brm, Snr1 or Mor favors the RA isoform relative to RC in S2 cells (Figure 6B). Figure 6C shows the validation of the result from Brm, and Figure 6E that of Mor. Depletion of Snr1 did not have the severe effect observed in the microarray experiments (compare Figure 6B with Figure 6D).

In summary, we identified a specific subset of transcripts that were affected by depletion of more than one SWI/SNF subunit and we could validate in S2 cells many of the results derived from the microarray experiments. These observations suggest that the levels of SWI/SNF complex, not of Brm alone, influence the processing of a subset of transcripts.

### Direct vs indirect effects of SWI/SNF levels on pre-mRNA processing

SWI/SNF is known to act as a transcriptional regulator and, if depletion of SWI/SNF subunits inhibited transcription, the changes observed in the levels of alternative transcripts could be due to differential stability. To investigate this possibility, we measured the global mRNA levels of the four selected genes (Additional file 1, Table S4) and the decay of the alternative transcripts (Table 2). For the analysis of the stability of the transcripts, Brm and Snr1 were knocked down in S2 cells, the cells were treated with actinomycin D, and the half-life times of the transcript variants were measured by RT-qPCR. The compilation of the results obtained in three independent knock-down experiments is shown in Table 2. For two of the genes, *CG3884 *and *mod(mdg4)*, the global mRNA levels were clearly reduced upon depletion of SWI/SNF subunits. However, the alternative transcripts showed similar half-life times under control conditions (GFP control samples).

**Table 2:** Analysis of transcript stability in S2 cells

Gene	Transcript	RNAi	Half-life time^1^	p-value^2^
*Gpdh *	RA	Brm	1.84 ± 0.41	0.31
		GFP	1.49 ± 0.11	
		SNR1	1.98 ± 0.31	0.09
*Gpdh *	RB	Brm	3.32 ± 1.33	0.12
		GFP	1.39 ± 0.16	
		SNR1	1.73 ± 0.24	0.12
*Gpdh *	RC	Brm	2.32 ± 0.20	0.02
		GFP	1.58 ± 0.17	
		SNR1	2.01 ± 0.22	0.03

*CG3884*	RA	Brm	1.47 ± 0.20	0.43
		GFP	1.30 ± 0.13	
		SNR1	1.40 ± 0.07	0.16
*CG3884*	RB	Brm	1.64 ± 0.11	0.32
		GFP	1.55 ± 0.02	
		SNR1	1.64 ± 0.34	0.67

*lola*	RA	Brm	1.41 ± 0.16	0.40
		GFP	1.92 ± 0.62	
		SNR1	1.47 ± 0.30	0.15
*lola*	RF	Brm	1.83 ± 0.15	0.10
		GFP	1.39 ± 0.12	
		SNR1	1.63 ± 0.04	0.11

*mod(mdg4)*	RA	Brm	1.59 ± 0.31	0.38
		GFP	1.30 ± 0.15	
		SNR1	1.35 ± 0.14	0.80
*mod(mdg4)*	RC	Brm	1.67 ± 0.47	0.29
		GFP	1.29 ± 0.04	
		SNR1	1.60 ± 0.13	0.05

^1^S2 cells were treated with dsRNA for Brm, GFP (control) or Snr1 for 24 hours, and treated with actinomycin D. RNA was purified after 0, 1.5 and 2.5 hours of treatment and quantified by RT-qPCR. The values are averages and standard deviations from three independent knock-down experiments.

^2 ^A paired, double-tailed Student's T-test was applied to compare the stabilities of each transcript in depleted and control cells.

We also analyzed whether the depletion of SWI/SNF subunits had any impact on the stability of the alternative mRNAs. In most cases, depletion of SWI/SNF subunits did not affect the stability of the transcripts, being *Gpdh-RC *an exception. Indeed, *Gpdh-RC *was significantly stabilized by depletion of either Brm or Snr1 (Table 2). In all other cases, however, the decay rates were not affected by the levels of SWI/SNF. In summary, the analysis of the decay rates indicated that differences in the stability of the alternative transcripts cannot account for the changes observed upon depletion of SWI/SNF subunits.

In another series of experiments, we asked whether Brm, Snr1 and Mor were associated with the *Gpdh*, *CG3884*, *lola *and *mod(mdg4) *genes, and we carried out chromatin immunoprecipitation (ChIP) experiments to answer this question. We could detect the SWI/SNF subunits associated with each of the genes but not with an intergenic sequence used as a negative control (Additional file 2, Figure S2). These observations suggest that the effects of SWI/SNF depletion on pre-mRNA processing were direct.

### The level of SWI/SNF affects pre-mRNA processing *in vivo*

**Figure 7:**
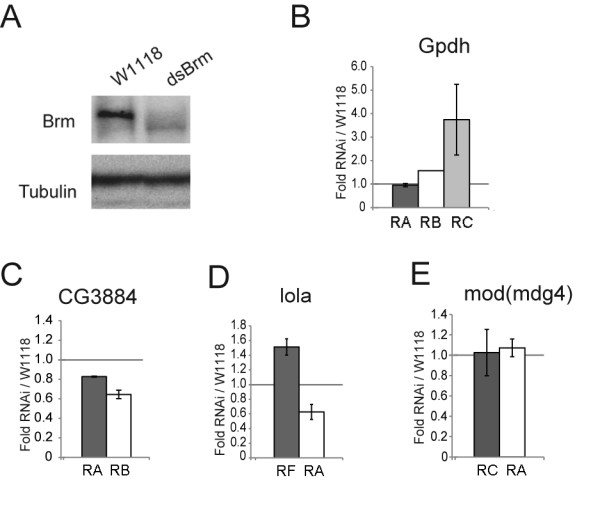
**Depletion of Brm by RNAi in vivo affects the relative abundances of alternative transcripts from the Gpdh and lola genes in larvae**. hs-GAL4 virgin females were crossed with UAS-BrmRNAi males, and early third instar larvae from the cross were heat shocked for 2 h at 37°C. Control experiments were carried out in parallel by crossing hs-GAL4 virgin females with wild type males W1118. (A) The effect of the depletion was analyzed 24 h after the heat shock by Western blotting using an anti-Brm antibody. The same blot was probed in parallel with an anti-tubulin antibody that served as a loading control. (B-E) hs-GAL4 virgin females were crossed with UAS-BrmRNAi males, the third instar larvae from the cross were heat shocked as above, and total RNA was isolated 24 h after the heat shock. Total RNA was purified and the abundances of selected transcripts were analyzed by RT-qPCR using the same primers as in Figures 3-6. In (B), (C) and (E), the relative abundance of each alternative transcript was calculated relative to the abundance of a constitutive exon. In (D), the relative abundance of each alternative transcript was calculated relative to the abundance of the Act5C mRNA, as in Figure 5. The results are expressed as average factor change between BrmRNAi and W1118 flies. The bars represent averages and the error bars represent standard deviations from two independent experiments, each quantified in duplicate (n = 4).

The experiments presented above were carried out using S2 cells. We next investigated whether SWI/SNF affects pre-mRNA processing also *in vivo*. For this purpose, we knocked down expression of *D. melanogaster *Brm using a systemic heat shock. We knocked down Brm *in vivo *by treating the larvae for 2 hours at 37°C, and we analyzed the RNAi effects 24 hours after the heat-shock treatment. The efficiency of the knockdown after heat shock was assessed by Western blotting using an anti-Brm antibody, as shown in Figure 7A. Total RNA was purified from total larva, and the levels of selected transcripts were quantified by RT-qPCR using the same primer pairs as those indicated in Figures 3, 4, 5, 6. The results from two independent experiments are shown in Figure 7. Knockdown of Brm *in vivo *did not influence the relative abundances of the analyzed transcripts of two of the genes analyzed, *CG3884 *and *mod(mdg4)*. In contrast, the *in vivo *knockdown of Brm affected the relative abundances of the alternatively processed transcripts of *Gpdh *and *lola*, and the effects were very similar to the effects observed in S2 cells. This result was confirmed with a second Brm RNAi strain (results not shown). We conclude that the level of Brm influences alternative pre-mRNA processing *in vivo*.

## Discussion

### Brm works in conjunction with other components of the SWI/SNF complex in pre-mRNA processing

Previous studies have shown that Brm influences the alternative processing of a subset of pre-mRNAs in human and insect cell lines [22,24,25] but the mechanisms responsible for such regulation are not known. In the studies mentioned above, a functional link between the levels of Brm and the splicing outcome has been established after experimental alteration of the Brm levels in cultured cells, either by over-expression or by RNAi-mediated depletion. These studies focused on the Brm subunit. We have now extended the previous study and asked whether depletion of other SWI/SNF subunits also results in alterations of pre-mRNA processing. In addition to Brm, we have now analyzed Mor, Snr1, PB, Bap170 and Osa in *D. melanogaster *S2 cells. We have mined microarray data from Moshkin and coworkers [4] and looked for genes whose relative abundances between alternative transcripts are changed by the RNAi treatment in a different manner than the abundances in mock-treated samples. The number of genes affected was low, but many of the detected events could be validated. This small collection of validated genes is valuable for future mechanistic studies.

The reasons for the low number of genes affected could be partly technical. First, the data used was obtained from expression arrays that do not cover all the splicing variants of the transcriptome of *D. melanogaster*. Second, the variances in the datasets were relatively high and, for this reason, we chose to avoid false positives by establishing stringent criteria and discarding genes that did not show consistent results in the replicates. In spite of these limitations, we identified a total of 45 genes for which the pre-mRNA processing levels changed after depletion of SWI/SNF subunits. Depletion of different SWI/SNF subunits affected different genes with a statistically significant overlap, in particular for the core subunits of the SWI/SNF complex. Indeed, we identified a group of ten genes that, according to the microarray data, were affected by depletion of at least two different core subunits. In summary, our results show that depletion of other core subunits apart from Brm influences pre-mRNA processing. This conclusion agrees with observations in human cells, where Brm modulates the splicing of the TERT transcripts in concert with the mRNA-binding protein p54(nrb) [24]. In the same study, Ito and coworkers showed that p54(nrb) and core subunits of the SWI/SNF complex interact physically.

We have analyzed the decay of the transcripts affected by depletion of SWI/SNF subunits and we can rule out differential stability as a major cause for the differences observed in the relative abundances of alternative transcripts. Using ChIP, we have also shown that Brm, Snr1 and Mor are asociated with the genes affected. Altogether, these observations support the conclusion that the mechanism by which SWI/SNF affects pre-mRNA processing is direct and cotranscriptional.

### The role of chromatin in the regulation of pre-mRNA processing

Alternative splicing and polyadenylation are major sources of transcript diversity and proteomic diversity in higher eukaryotes. Complex regulatory networks determine the pre-mRNA processing outcome and play critical roles in differentiation and development. Key elements in such regulatory networks are the splicing and polyadenylation factors that influence the choice of alternative processing sites by binding to *cis*-acting elements, either enhancers or silencers, in the pre-mRNAs [27]. Recent research has revealed that, in addition to the RNA sequence itself, the chromatin environment and the transcription machinery contribute to the recruitment of regulatory factors to their target transcripts during transcription [reviewed by [28,29]]. Genome-wide studies have shown that certain histone modifications are non-randomly distributed in exons and introns [30,31], and that nucleosomes are enriched in exonic sequences [32,33]. The functional significance of these observations is not fully understood, but there are examples of adaptor proteins that bind both to splicing factors and to specific histone modifications, and such adaptors may play important roles in the targeting of regulatory factors to the pre-mRNA [34]. Another determinant of the splicing outcome is the elongation rate of RNAP II. A reduction of the RNAP II elongation rate at specific positions along the gene can facilitate the assembly of the splicing machinery at weak splice sites and promote the inclusion of proximal exons [reviewed by [35,36]]. hBrm regulates the alternative splicing of the CD44 pre-mRNA in human cells by decreasing the elongation rate of RNAP II and inducing the accumulation of the enzyme at specific positions in the gene [22]. In the case of the CD44 gene, Brm favors the usage of proximal processing sites. We have shown that in *D. melanogaster *the SWI/SNF complex regulates the processing of a subset of pre-mRNAs through somehow different mechanisms. In some of the cases that we have analyzed, depletion of SWI/SNF promotes the use of a proximal splice site (see, for instance, the up-regulation of the *lola-RA *transcript shown in Figure 5), which cannot be explained by the same mechanisms that act on the human CD44 gene. Several alternative mechanisms can be envisioned. In one scenario, SWI/SNF either decreases or increases the transcription rate, depending on the genomic context and on the presence of specific regulators. Alternatively, SWI/SNF could act by a mechanism that is independent from the transcription kinetics. We have previously shown that a fraction of SWI/SNF is associated with nascent transcripts [25], while other authors have shown that Brm and specific mRNA-binding proteins interact [22,24]. It is tempting to speculate that SWI/SNF plays a more direct role in pre-mRNA processing, possibly by modulating the recruitment and/or assembly of splicing or polyadenylation factors.

### SWI/SNF regulates pre-mRNA processing *in vivo*

Previous research on the role of Brm in pre-mRNA processing was carried out in cultured cells. We have now depleted Brm in larvae and detected changes in pre-mRNA processing *in vivo*. Depletion of Brm had no significant effect on two of the four genes analyzed, *CG3884 *and *mod(mdg4)*. Depletion of Brm *in vivo *affected *lola *and *Gpdh*, in contrast, in a similar manner to its effect in S2 cells. It is important to point out that we analyzed RNA extracted from total larvae, not from individual organs, which might have occluded tissue-specific effects. Indeed, the gene expression data in FlyAtlas http://www.flyatlas.org/ shows that the expressions of the analyzed genes vary among organs and throughout development. Analyzing total larvae gives an average of the effects in the entire organism, which might not reflect the physiological regulation of the target genes in any specific tissue. However, the fact that Brm depletion affects the processing of the *lola *and *Gpdh *transcripts in larvae shows that the reported effect of SWI/SNF on pre-mRNA processing is not an artefact that occurs only in cultured cells.

The *lola *and *Gpdh *genes are structurally very different. *Gpdh *is a relatively short gene with three alternative mRNAs that encode nearly identical proteins. The alternative processing of the *Gpdh *pre-mRNA determines the sequence of the 3' UTRs, which can have a profound impact on the stability of the transcripts, their regulation by microRNAs and their translational properties. The *lola *gene, in contrast, is very long with at least 26 different transcripts that code for a plethora of protein isoforms characterized by different types of DNA-binding motifs. Therefore, *in vivo *regulation of *lola *by SWI/SNF affects the abundances of protein isoforms with different biological activities.

## Conclusion

We have shown that SWI/SNF can modulate alternative pre-mRNA processing, not only in cultured cells but also *in vivo*. The effect is restricted to and specific for a subset of transcripts, both in S2 cells and in larvae. Our results provide novel insights into the mechanisms by which SWI/SNF regulates transcript diversity and proteomic diversity in higher eukaryotes.

## Methods

### Microarray analysis

The microarray data was extracted from Array Express, E-TABM-169 http://www.ebi.ac.uk/microarray-as/aew/. *Drosophila *Genome 2.0 Arrays (Affymetrix) were hybridized with total RNA purified from *Drosophila *S2 cells treated with dsRNA corresponding to Brm, Snr1, Mor, PB, Bap170, and Osa [4]. The E-TABM-169 data contains four biological replicates for each of the RNAi treatments and six mock replicates. All the replicates were included in our initial analysis. A second analysis was carried out with only three and four replicates for treated and mock cells, respectively, after discarding deviant datasets identified by cluster analysis. In all cases, we mined the E-TABM-169 data, selected those genes that were represented by more than one probe set in the *Drosophila *Genome 2 arrays (974 genes) and identified among them the genes expressed in S2 cells. In the analysis that included all the data sets, we selected as "expressed" those probe sets that scored "present" in at least four out of six individual mock replicates, and in three out of the four treated replicates. In the second data analysis, we selected probe sets that scored "present" in at least three out of four mock experiments and two out of three treated replicates. We calculated the average signals for each expressed probe set, and we discarded probe sets for which the ratio of average to standard deviation was below 1.25, in order to exclude probe sets with large variances. We then calculated pair-wise for each gene the ratios between probe-set average signals. We set the criterion that both probe sets in each comparison satisfy the cut-off values for present signals and experimental variance, as described above. We finally selected probe-set pairs that showed at least a 2-fold ratio difference in the RNAi-treated experiments compared to mock. We used the gene models available at FlyBase [37] and the probe-set annotations at Affymetrix to analyze the structure of the genes selected. Gene ontology enrichment analyses were performed using the GOEAST software at http://omicslab.genetics.ac.cn/GOEAST/index.php

### Animals and cell culture

*Drosophila melanogaster *S2 cells were cultivated at 28°C in Schneider's medium (Invitrogen) supplemented with 10% heat-inactivated fetal bovine serum, 50 μg/ml penicillin and 50 μg/ml streptomycin. *Drosophila melanogaster *RNAi lines from the Vienna Drosophila RNAi Center were used. To account for differences in knockdown due to position effects, we used two available lines (#37720 and #37721) for Brm (CG5942). *W^1118 ^*and *hs-GAL4 *lines were obtained from the Bloomington stock center. All stocks were maintained on a standard cornmeal/molasses medium at 25°C.

### RNA interference in S2 cells

Double-stranded RNAs complementary to Brm, Snr1, Mor, and GFP were synthesised by *in vitro *transcription (MegaScript RNAi kit, Ambion) from gene-specific PCR fragments with T7 promoters incorporated at both ends. The sequences of the primers used are specified in the Additional file 1, Table S5. 3 × 10^6 ^S2 cells were cultured in 6-well plates over-night, washed with serum-free and antibiotic-free Schneider's medium, and treated with 30 μg of dsRNA/well. Cells treated with dsRNA complementary to Brm and SNR1 were harvested at 24 and 48 hours, respectively, after dsRNA addition. A second administration with dsRNA against Mor was carried out at 48 hours, and the cells harvested 96 hours after administration.

### RNA interference in *Drosophila melanogaster *larvae

*hs-GAL4 *virgin females were crossed with *UAS-BrmRNAi *males (strain #37721), and early third instar larvae from the cross were heat shocked for 2 h at 37°C. Control experiments were carried out in parallel by crossing *hs-GAL4 *virgin females with wild type males *W^1118^*. 24 h after the heat shock, late third instar larvae were collected for Western blot assay and RT-qPCR. Total RNA was extracted from ten larvae with Trizol according to the protocol provided by Invitrogen. cDNA synthesis was performed as described below. The results obtained with strain #37721 were confirmed using a second Brm RNAi strain, #37720.

### RNA extraction and quantitative RT-PCR

Total RNA was extracted from cells using the RNAqueous kit (Ambion) and DNase-treated with Turbo DNA-free (Applied Biosystems) according to the protocols provided by the manufacturers. Subsequently, cDNA synthesis was performed with 1 μg total RNA using Superscript III (Invitrogen). Quantitative PCR measurements were carried out in triplicate using a KAPA SYBR Fast qPCR Kit (KAPA Biosystem) in a QIAGEN Rotor-Gene Q system. Transcript levels were analyzed with isoform-specific primers (Table S5). The relative abundance of each transcript is presented as fold change compared to the reference sample, GFP for S2 cells and *W^1118 ^*for larvae, after normalizing the values obtained for each alternative transcript either to a common exon in the same transcript or to Actin 5C.

### Analysis of RNA decay

RNA interference was carried out in S2 cells as previously described using dsRNA for Brm and Snr1, and subsequently the cells was treated with 2 μg/ml Actinomycin D for 0, 1.5 and 2.5 hours. RNA levels were quantified by RT-qPCR with isoform-specific primers (Table S5) and normalized to ribosomal 28S RNA levels.

### ChIP

ChIP was performed essentially as described by Takahashi et al. (2000) with some modifications (Hessle et al., 2009). *Drosophila *S2 cells were fixed at room temperature with 2% formaldehyde. The immunoprecipitated DNA was purified and analyzed by PCR using gene-specific primers (Table S5).

### SDS-PAGE and Western blotting

Cell pellets were resuspended in SDS-PAGE sample buffer supplemented with 8 M urea and heated to 110°C. Larvae were ground in the same sample buffer, heated to 100°C for 10 min, vortexed, re-heated to 100°C and kept at this temperature for 5 min. Proteins were separated by SDS-PAGE using the Mini-Protean II system (BioRad) and transferred to polyvinylidenefluoride (PVDF) membranes (Millipore) in Tris-glycine buffer with 0.02% SDS and 4 M urea using a semi-dry electrophoretic transfer cell (BioRad). The antibodies were diluted in 0.05% Tween-20 and 1% milk in PBS and antibody incubations were carried out following standard procedures. The ECL system (GE Healthcare) was used for chemiluminiscent detection of horseradish peroxidase.

### Antibodies

The anti-Brm antibody was raised in rabbit and has been characterized in a previous study (Tyagi et al. 2009). The antibodies against Mor and Snr1 were kindly provided by C.P. Verrijzer (Moshkin et al. 2007 and references therein). The monoclonal anti-alpha-tubulin antibody (clone B-5-1-2) was from Sigma-Aldrich.

## Competing interests

The authors declare that they have no competing interests.

## Authors' contributions

JW, AKÖ and NV conceived the study. JW carried out the bioinformatic analysis of the microarray data together with DB, the knockdown of SWI/SNF subunits in S2 cells, the Western blot analyses and the ChIP experiments. JW also carried out the RNA stability experiments together with SY. JW wrote the initial draft the manuscript. ZW carried out the RNAi experiments in flies under the supervision of UT. AT contributed to the RNAi experiments in S2 cells. DB contributed to the bioinformatic analyses. AKÖ participated in the design of the study and in the discussion of the results. NV coordinated the study and drafted the final version of the manuscript. All authors read and approved the final manuscript.

## Supplementary Material

Additional file 1 **Tables S1-S5**. Table S1: List of genes for which depletion of SWI/SNF subunits affects the relative abundances between alternative transcripts originated by alternative pre-mRNA processing. Table S2: List of genes for which depletion of SWI/SNF subunits affects the relative abundances between alternative transcripts transcribed from different promoters. Table S3: Microarray expression signals for selected transcripts from Array Express E-TABM-169. Table S4: Global mRNA levels for *Gpdh*, *lola*, *mod(mdg4) *and *CG3884 *analyzed by RT-qPCR. Table S5: The sequences of the primers used for synthesis of dsRNA and for RT-qPCR. Click here for file

Additional file 2 **Figures S1 and S2**. Figure S1: Simultaneous depletion of SWI/SNF signature subunits. The figure compares the effects of Brm depletion with those of simultaneous depletion of Osa, Pb and Bap170 on the relative abundances of *lola *and *CG3884 *transcripts. Figure S2: The figure shows ChIP experiments to analyze the association of Brm, Mor and Snr1 with the selected genes. Click here for file
