# Supplementary material for: SWI/SNF regulates the alternative processing of a specific subset of pre-mRNAs in Drosophila melanogaster
Source: BMC Mol Biol. 2011 Nov 2;12:46. doi: 10.1186/1471-2199-12-46 (PMC3221629; doi:10.1186/1471-2199-12-46)
Supplement: Additional file 1 — Tables S1-S5. Table S1: List of genes for which depletion of SWI/SNF subunits affects the relative abundances between alternative transcripts originated by alternative pre-mRNA processing. Table S2: List of genes for which depletion of SWI/SNF subunits affects the relative abundances between alternative transcripts transcribed from different promoters. Table S3: Microarray expression signals for selected transcripts from Array Express E-TABM-169. Table S4: Global mRNA levels for Gpdh, lola, mod(mdg4) and CG3884 analyzed by RT-qPCR. Table S5: The sequences of the primers used for synthesis of dsRNA and for RT-qPCR. [file 1471-2199-12-46-S1.DOC]

**Table S1**

**List of genes for which depletion of SWI/SNF subunits affects the relative abundances between alternative transcripts originated by alternative pre-mRNA processing**

alph

Arpc3A

CalpA

CG10077

CG10638

CG13784

CG16890

CG17838

CG2051

CG32495

CG33969

CG3884

CG3964

CG4662

CG5174

CG6791

CG7920

CG7971

CG8092

CG8177

CG8290

dl

faf

fs(2)ltoPP43

Gpdh

Gs1l

Hairless

Idgf3

l(1)G0196

lola

mbl

Mmp1

Mod (mdg4)

mud

Pabp2

Pyd

Rbp1

RpS11

Sap47

Smp-30

Sno strawb.

spir

sqd

sucb

Trl GaGa

**Table S2**

**List of genes for which depletion of SWI/SNF subunits affects the relative abundances between alternative transcripts transcribed from different promoters**

CG33054

CG6900

Ald

Atpalpha

capt

CG10962

CG17838

CG2225

CG32626

CG6854

cp309

jog

Eip74EF

fru

grp

H

larp

mew

mld

norpA

RpL35

RpS11

RpS30

wdb

shn

Ect4

gfzs

**Table S3**

Microarray expression signals for selected transcripts from Array Express E-TABM‑169 (Moshkin et al. 2007). The table gives the average signals, standard deviations and the ratios of the average to the standard deviation for each of the selected probe sets.

|  |  |  |  |  |  |  |  |  |  |  |  |
| --- | --- | --- | --- | --- | --- | --- | --- | --- | --- | --- | --- |
|  |  |  | **Control (mock-RNAi)** | | | **BRM-RNAi** | |  | **SNR1-RNAi** | |  |
| Probe set ID | Gene | Transcript | avg | stdev | avg/stdev | avg | stdev | avg/stdev | avg | stdev | avg/stdev |
| 1636311_at | Gpdh | RA | 151.7 | 99.9 | 1.5 | 55.4 | 14.1 | 3.9 | 53.8 | 28.3 | 1.9 |
| 1625949_at | Gpdh | RB | 305.5 | 128.5 | 2.4 | 214.3 | 12.2 | 17.5 | 203.1 | 60.9 | 3.3 |
| 1634893_at | Gpdh | RC | 13.2 | 1.5 | 8.6 | 25.2 | 6.9 | 3.7 | 17.3 | 2.1 | 8.4 |
| 1638816_at | CG3884 | RA | 192.6 | 59.9 | 3.2 | 69.1 | 8.3 | 8.3 | 72.9 | 28.2 | 2.6 |
| 1628581_at | CG3884 | RB | 495.3 | 292.4 | 1.7 | 56.2 | 25.0 | 2.2 | 83.7 | 57.6 | 1.5 |
| 1634052_s_at | mod(mdg4) | RA | 83.2 | 38.2 | 2.2 | 20.8 | 9.6 | 2.2 | 24.8 | 7.4 | 3.4 |
| 1631833_at | mod(mdg4) | RC | 229.6 | 68.6 | 3.3 | 187.0 | 34.1 | 5.5 | 156.3 | 33.9 | 4.6 |
| 1641609_at | lola | RA | 51.2 | 8.7 | 5.9 | 73.4 | 6.2 | 11.9 | 70.8 | 5.4 | 13.1 |
| 1628946_at | lola | RF | 65.5 | 27.8 | 2.4 | 46.3 | 8.5 | 5.5 | 29.0 | 14.6 | 2.0 |

|  |  |  |  |  |  |  |  |  |  |  |  |
| --- | --- | --- | --- | --- | --- | --- | --- | --- | --- | --- | --- |
|  |  |  | **MOR-RNAi** | |  | **OSA-RNAi** | |  | **PB-RNAi** | |  |
| Probe set ID | Gene | Transcript | avg | stdev | avg/stdev | avg | stdev | avg/stdev | avg | stdev | avg/stdev |
| 1636311_at | Gpdh | RA | 38.2 | 14.5 | 2.6 | 75.1 | 16.5 | 4.6 | 112.3 | 27.6 | 4.1 |
| 1625949_at | Gpdh | RB | 209.0 | 88.8 | 2.4 | 221.4 | 18.6 | 11.9 | 153.4 | 68.7 | 2.2 |
| 1634893_at | Gpdh | RC | 18.7 | 12.9 | 1.4 | 7.4 | 5.4 | 1.4 | 15.8 | 6.8 | 2.3 |
| 1638816_at | CG3884 | RA | 54.4 | 16.5 | 3.3 | 205.0 | 42.7 | 4.8 | 84.1 | 20.2 | 4.2 |
| 1628581_at | CG3884 | RB | 63.7 | 30.5 | 2.1 | 355.8 | 106.0 | 3.4 | 134.9 | 41.8 | 3.2 |
| 1634052_s_at | mod(mdg4) | RA | 19.1 | 2.6 | 7.4 | 25.0 | 24.2 | 1.0 | 46.8 | 24.3 | 1.9 |
| 1631833_at | mod(mdg4) | RC | 185.8 | 69.0 | 2.7 | 169.9 | 3.7 | 45.3 | 216.1 | 57.1 | 3.8 |
| 1641609_at | lola | RA | 61.0 | 15.0 | 4.1 | 77.0 | 19.8 | 3.9 | 50.8 | 14.9 | 3.4 |
| 1628946_at | lola | RF | 53.9 | 24.7 | 2.2 | 51.9 | 15.1 | 3.4 | 45.0 | 7.0 | 6.5 |

|  |  |  | **BAP170-RNAi** | |  | **OSA_PB_BAP170-RNAi** | | |
| --- | --- | --- | --- | --- | --- | --- | --- | --- |
| Probe set ID | Gene | Transcript | avg | stdev | avg/stdev | avg | stdev | avg/stdev |
| 1636311_at | Gpdh | RA | 51.2 | 20.6 | 2.5 | 57.0 | 18.2 | 3.1 |
| 1625949_at | Gpdh | RB | 128.2 | 40.5 | 3.2 | 150.0 | 34.8 | 4.3 |
| 1634893_at | Gpdh | RC | 11.5 | 2.1 | 5.5 | 10.4 | 2.6 | 4.1 |
| 1638816_at | CG3884 | RA | 112.3 | 38.1 | 2.9 | 42.4 | 9.1 | 4.7 |
| 1628581_at | CG3884 | RB | 206.2 | 69.6 | 3.0 | 36.3 | 23.8 | 1.5 |
| 1634052_s_at | mod(mdg4) | RA | 32.9 | 5.8 | 5.7 | 44.2 | 10.2 | 4.3 |
| 1631833_at | mod(mdg4) | RC | 111.3 | 23.8 | 4.7 | 144.8 | 25.9 | 5.6 |
| 1641609_at | lola | RA | 52.2 | 11.3 | 4.6 | 68.8 | 11.3 | 6.1 |
| 1628946_at | lola | RF | 33.8 | 9.1 | 3.7 | 33.7 | 8.5 | 4.0 |

**Table S4**

Global mRNA levels analyzed by RT-qPCR. The primers used target the constitutive exons indicated in the figures. For lola, the left primer targets the junction between exons 1 and 2, and the left primer targets exon 2. The table gives fold expression compared to GFP controls.

| **Gene** | **Brm** | **Mor** | **SNR1** |
| --- | --- | --- | --- |
| Gpdh | 1.10 | 0.86 | 0.86 |
| CG3884 | 0.55 | 0.64 | 0.82 |
| mod(mdg4) | 0.63 | 0.70 | 0.75 |
| lola | 1.62 | 0.79 | 1.49 |

| **Table S5** | | |  | |  | |  |  | | | | | |  |
| --- | --- | --- | --- | --- | --- | --- | --- | --- | --- | --- | --- | --- | --- | --- |
| Sequences of the primers used for synthesis of dsRNA and for RT-qPCR | | | | | | | | | | | | | |  |
|  |  | | |  | |  |  | |  | | | | | |
| **Primers used for synthesis of dsRNA** | | | | | |  |  | |  | | | | | |
| Brm-BKNT7-F | | taatacgactcactatagggagaAAGCCCAATCGCATTACAAC | | | | | | | | | | |  | |
| Brm-BKNT7-R | | taatacgactcactatagggagaTGAACTGTATCAGCCGCTTG | | | | | | | | | | |  | |
| Brm-HFAT7-F | | TTAATACGACTCACTATAGGGAGAgtttcgctgtacaataacaatc | | | | | | | | | | | | |
| Brm-HFAT7-R | | TTAATACGACTCACTATAGGGAGAatgtggagcaggacttaaag | | | | | | | | | | |  | |
| SNR1_HFA F | | taatacgactcactatagggagaGGGAGAGCAAGTCAAAGAAG | | | | | | | | | | |  | |
| SNR1_HFA R | | taatacgactcactatagggagaCTTCTCGGACATGTCCCAC | | | | | | | | | | |  | |
| Mor_HFA F | | taatacgactcactatagggagaAGTACACGCCTTCTTGGAG | | | | | | | | | | |  | |
| Mor_HFA R | | taatacgactcactatagggagaCAGAATGCACTCGTCTTGA | | | | | | | | | | |  | |
| Mor DRSCT7 F | | taa tac gac tca cta tag gga ga CGAGATCCTGGACAATCGAC | | | | | | | | | | | | |
| Mor DRSCT7 R | | taa tac gac tca cta tag gga ga GATCGGGCAGGTCAAAGTTA | | | | | | | | | | | | |
|  | |  | | | | | | | |  |  |  |  | |
| **Primers used for RT-qPCR** | | | | | | | | | |  |  |  |  | |
| Gdph-RA Fw | | GGTCTGGAGGACAAATTCCCCCTGT | | | | | | | |  |  |  |  | |
| Gdph-RA Rev | | CGGGGGATTTTCGGGAAGGCA | | | | | | | |  |  |  |  | |
| Gdph-RB Fw | | ACGCAATCACCCTGAGCATATGGAT | | | | | | | |  |  |  |  | |
| Gdph-RB Rev | | AGGCCTTGGTGAAGATGACTGGT | | | | | | | |  |  |  |  | |
| Gdph-RC Fw | | GGTCTGGAGGACAAATTCCCCCTGT | | | | | | | |  |  |  |  | |
| Gdph-RC Rev | | TCGAGTTGGCGTTGTGCTGAA | | | | | | | |  |  |  |  | |
| Gpdh seq_372_F | | AATCGCGGAGCCAAGTAGTA | | | | | | | | |  |  |  | |
| Gpdh const R | | AGCCCACAATGCACACATTT | | | | | | | |  |  |  |  | |
| CG3884 RB 4 F | | CCTGCCCCCATACGCGGTTA | | | | | | | | |  |  |  | |
| CG3884 RB 4 R | | ATAGAGCGGCTCGCCCGTT | | | | | | | | |  |  |  | |
| CG3884_RA 3 F | | GTGGTGGGCGGCAACGATGA | | | | | | | | |  |  |  | |
| CG3884_RA 3 R | | TGCTCGGCACCACCTTGCAG | | | | | | | | |  |  |  | |
| CG3884 F | | CAAACTTTGGTCAGTTCAGACG | | | | | | | | |  |  |  | |
| CG3884 R | | TCACTTGCGTTCTTCTGACTCC | | | | | | | | |  |  |  | |
| lola_RA F | | ACGGGATCCCCAAGGCCTGC | | | | | | | | |  |  |  | |
| lola_RA R | | CCCCCATTTCCTGCTCCGCC | | | | | | | | |  |  |  | |
| lola_RF F | | GCAACGGGATCCCCAAGCTCC | | | | | | | | |  |  |  | |
| lola_RF R | | TCAGGGCTTGCGCCGAACTG | | | | | | | | |  |  |  | |
| lola_const F | | CACAACACATAACTGCTGACACA | | | | | | | | |  |  |  | |
| lola_const R | | GCAAAGTAGGGACTGCATGC | | | | | | | | |  |  |  | |
| Mod(mdg4)-all F | | CACCCACGCTATCGTATTCC | | | | | | | | |  |  |  | |
| Mod(mdg4)-all R | | CGTTAGCCCCTTGATTTGC | | | | | | | | |  |  |  | |
| Mod(mdg4)-RA F | | GGGCAACACAGAGGCTCAAGAAAA | | | | | | | | | |  |  | |
| Mod(mdg4)-RA R | | GGTGTACTGTGTGCAGCGCCA | | | | | | | | |  |  |  | |
| Mod(mdg4)-RC F | | AGGCTCAAGATGGCCCAAGCA | | | | | | | | |  |  |  | |
| Mod(mdg4)-RC R | | GACCCGGCTGCCATCGAACA | | | | | | | | |  |  |  | |
| Actin 2 F | | GCACACCCACAAGCTTACACA | | | | | | | | |  |  |  | |
| Actin 2 R | | TTGCGCTTTGGGAAATATCTTC | | | | | | | | | |  |  |  |
| 28s ae30 | | ATGACGAGGCATTTGGCTAC | | | | | | | | |  |  | | |
| 28s ae29 | | CCCAGTGCTCTGAATGTCAA | | | | | | | | | |  | | |

Primers used for ChIP analysis

| Inter-R | AACAGTTTGTTTGGCCCTTG |
| --- | --- |
| Inter-F | ACTCCCACTCACACGGAA AC |
| CG9380 Prox F | GGTGTGGTAGGATGCGGTAG |
| CG9380 Prox R | TGAAACTTTGCAATCGGTGA |
| lola-RA F4 | GCGATGACTATCACCAGGGA |
| lola-RA R4 | ACTATTTCCGCCGGCTAATAA |
| Mod(mdg4)-RY R | CTCTGCTTGCGGGAGCCACC |
| 5UTR RY Intron F | TTACTTGGCGGATCCTCTTG |
| Gpdh seq_372_F | AATCGCGGAGCCAAGTAGTA |
| Gpdh const R | AGCCCACAATGCACACATTT |
| CG3884 F | CAAACTTTGGTCAGTTCAGACG |
| CG3884 R | TCACTTGCGTTCTTCTGACTCC |
