# Supplementary material for: SWI/SNF regulates the alternative processing of a specific subset of pre-mRNAs in Drosophila melanogaster
Source: BMC Mol Biol. 2011 Nov 2;12:46. doi: 10.1186/1471-2199-12-46 (PMC3221629; doi:10.1186/1471-2199-12-46)
Supplement: Additional file 2 — Figures S1 and S2. Figure S1: Simultaneous depletion of SWI/SNF signature subunits. The figure compares the effects of Brm depletion with those of simultaneous depletion of Osa, Pb and Bap170 on the relative abundances of lola and CG3884 transcripts. Figure S2: The figure shows ChIP experiments to analyze the association of Brm, Mor and Snr1 with the selected genes. [file 1471-2199-12-46-S2.DOC]

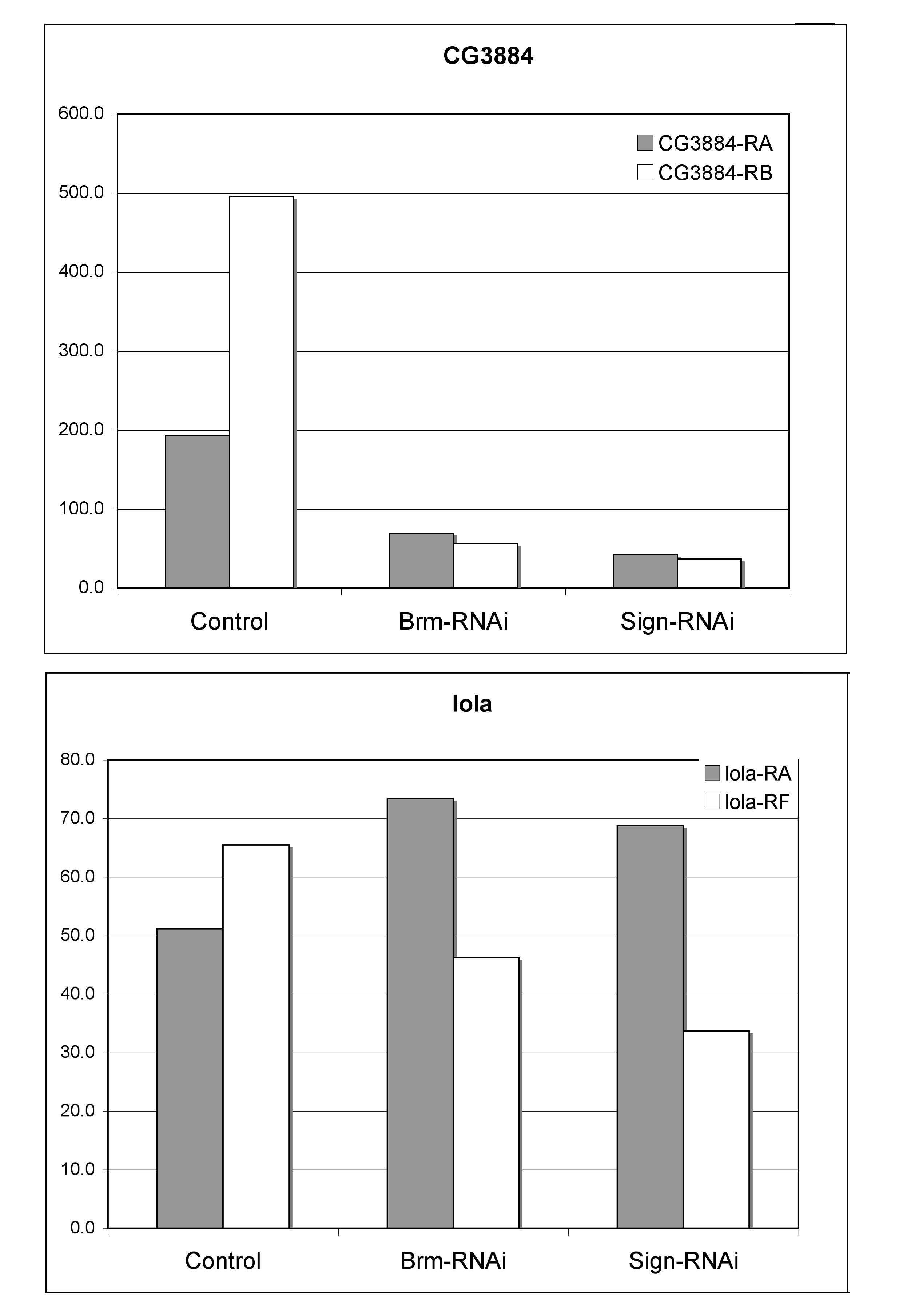


**Figure S1.** Effect of the simultaneous depletion of SWI/SNF signature subunits on pre-mRNA processing.

The figure compares the effects of Brm depletion with those of simultaneous depletion of Osa, Pb and Bap170 on the relative abundances of *lola* and *CG3884* transcripts.

Based on microarray data from ArrayExpress E-TABM-169.

**Figure S2.** The association of Brm, Mor and Snr1 with the selected genes analyzed by ChIP.

Chromatin was extracted from S2 cells after fixation with formaldehyde, and immunoprecipitation reactions were carried out with antibodies against Brm, Mor and Snr1, as indicated in the figure. A negative control immunoprecipitation was carried out in parallel (*neg*). The immunoprecipitated DNA was analyzed by PCR using primers specific for *Gpdh*, *CG3884*, *lola* and *mod(mdg4).* As a negative control, an intergenic region devoid of annotated genes was analyzed in parallel. The *CG9380* gene was used as a postive control (Tyagi et al., 2009).
